# The Lack of Relationship between an Endothelin-1 Gene Polymorphism (Ala288Ser) and Incidence of Hypertension: A Retrospective Cohort Study among Japanese Workers

**DOI:** 10.2188/jea.14.129

**Published:** 2005-03-18

**Authors:** Akihiko Kaetsu, Takuji Kishimoto, Yoneatsu Osaki, Mikizoh Okamoto, Soji Fukumoto, Yoichi Kurozawa

**Affiliations:** 1 Division of Environmental and Preventive Medicine, Faculty of Medicine, Tottori University.; 2 Division of Molecular Medical Zoology, Faculty of Medicine, Tottori University.; 3 Division of Health Administration and Promotion, Faculty of Medicine, Tottori University.

**Keywords:** endothelin-1, polymorphism (genetics), hypertension, cohort studies, Japanese workers

## Abstract

BACKGROUND: Some case-control association studies revealed the relationship between some endothelin-1 (ET-1) gene polymorphisms and blood pressure. Because no report was available about the relationship between any ET-1 gene polymorphism and incidence of hypertension, we examined the relationship between novel ET-1 gene polymorphism (G862T / Ala288Ser in exon 5) and incidence of hypertension by a retrospective cohort study.

METHODS: The subjects were Japanese workers at a company in Shimane Prefecture in Japan. The polymorphism with genome DNA extracted from the blood of the workers was analyzed using the polymerase chain reaction confronting two pair primers method. According to the results of two regular health checkups with a 6-year interval, the study population was divided into two groups by blood pressure and antihypertensive treatment in 1998, after excluding people who had hypertension in 1992.

RESULTS: There were 133 (93 males and 40 females) incidences of hypertension observed among the study population of 922 (540 males and 382 females). In the univariate analysis, odds ratios o Ala/Ser and Ser/Ser against Ala/Ala were 0.98 (95% confidence interval [CI]): 0.7-1.4) and 0.79 (95% CI: 0.4-1.6), respectively. In the multivariate analysis adjusted for sex, age, body mass index, serum total cholesterol, fasting blood sugar, and smoking and drinking habits, odds ratios for Ala/Ser and Ser/Ser against Ala/Ala were 0.97 (95% CI: 0.7-1.4) and 0.75 (95% CI: 0.4-1.5), respectively.

CONCLUSIONS: The ET-1 gene polymorphism in this study did not seem to be associated with the incidence of hypertension among the Japanese workers.

Endothelin (ET) is known as the most potent vasoconstrictor among reported endothelial-derived vasomotor agents.^1^^,^^2^ Among the reported 3 isoforms of ET, endothelin-1 (ET-1) is produced by endothelial cells.^3^ ET-1 plays a role not only in vasoconstriction but also in vasodilatation through induction of an endothelial cell-derived relaxing factor, nitric oxide.^4^ These functions of ET-1 correlate to the pathogenesis of atherosclerosis.^5^ Thus, the association between ET-1 and hypertension- or atherosclerosis-related diseases has been studied. In fact, increased plasma ET-1 level has been demonstrated among pre-eclamptic pregnant women,^6^ patients with hypertension^7^ and diabetes,^8^ respectively. Furthermore, because a reduction effect on blood pressure with the induction of ET receptor antagonist was observed,^9^^,^^10^ the role of ET-1 in regulation of human blood pressure was confirmed.

The human ET-1 gene was assigned to chromosome 6, and spans approximately 5.5 kb with 5 exons and 4 introns.^11^^,^^12^ Some epidemiologic studies analyzing the relationship between some polymorphisms in this locus and hypertension have been performed as case-control association studies.^6^^,^^13^^-^^18^ Moreover, the relationship between a polymorphism in the ET-1 gene and ventricular arrhythmia has been reported.^19^

Although the presence of a novel polymorphism, the G/T polymorphism with an amino acid substitution (Ala to Ser) at codon 288 in exon 5 at position 862 of the ET-1 gene (Ala288Ser), was released,^20^ the prevalence of the polymorphism among general population was not available. In this way, the presence of a novel human ET-1 gene polymorphism was revealed, but the relationship between the polymorphism and hypertension was not available.

Thus, in the present study, we analyzed the relationship between the novel ET-1 gene polymorphism and hypertension among Japanese workers. To assess the relationship more detail than in previous reports, a retrospective cohort study enrolling lifestyle factors as considerable variables was performed.

## METHODS

### Study population and eligible cohort subjects

The study population was recruited from workers of a company who received annual health checkups and gave written informed consent in 1998, in Shimane Prefecture in Western Japan. The total number of the study population was 1,978. Among the study population of our study, 1,175 subjects were received annual health checkups in both 1992 and 1998. The number of subjects who assessed as normotensive was 975, after excluding hypertensive in 1992, sat as a study cohort. Because there were 922 subjects whose various variables for this study were known, 95% of normotensives in 1992 were enrolled in this study as eligible cohort subject. The incidence of hypertension was assessed at the health checkups in 1998. The following selection criteria for hypertension were applied both in 1992 and 1998: (1) hypertension was defined by systolic blood pressure (SBP) of 140 mmHg and over and/or diastolic blood pressure (DBP) of 90 mmHg and over; or (2) subject received medication for hypertension. The eligible subjects were divided into two groups by incidence of hypertension (i.e., hypertensive and normotensive in 1998). The number of subjects who developed the hypertension in 1998 was 133, 14.4% of the eligible subjects. Among the 133 incident cases, 128 subjects were assessed based on their blood pressure in 1998. Further, 111 subjects showed SBP less than 160 mmHg and/or DBP less than 100 mmHg.

### Various measurements and survey of life habits

Blood pressure was measured with the subjects seated, on the right arm, using a standard mercury sphygmomanometer after at least 5 minutes of rest subsequent to urination. Phase I and phase V Korotkov sounds were recorded as SBP and DBP, respectively. Blood pressure was measured once for each subject, when SBP was less than 140 mmHg and DBP was under 90 mmHg. In case of SBP with 140 mmHg and higher and/or DBP with 90 mmHg and higher, re-measure was performed after at least 5 minute interval. Then the lower of the two measurements was used as the blood pressure. Although this method is not common for epidemiologic studies, it is common for health checkups for workers. The purpose of this method is to evaluate whether each subject has hypertension or not. Further, this method is based on the recommendation from the Japanese Association for Cerebro-cardiovascular Disease control,^21^ basically. Because it seemed to provide the information of which equal to use the second of two measurements and better than use the mean of two measurements, this method was employed for our studies.

Body Mass Index (BMI), body weight in kilogram divided by squared height in meter, was used as a measure of body composition.

Blood samples were drawn into disposable plastic vacuum tubes (Becton, sst gel and clot activator) from a peripheral vein. Serum total cholesterol (TC) and fasting blood sugar (FBS) were measured using an autoanalyzer (Model 7150 Autoanalyzer, Hitachi). TC and FBS were determined enzymatically using commercial enzyme kits (cholesterol oxidase method, Kyowa Medex, Tokyo, Japan, and Hexokinase glucose 6 phosphatedehydrogenase method, Wako, Tokyo, Japan, respectively).

Alcohol drinking and smoking habits were assessed using a questionnaire on lifestyle factors.

### Endothelin-1 gene polymorphism Ala288Ser

The ET-1 gene polymorphism studied here was selected from released polymorphisms^20^ according to variation in the exon loci where the protein is encoded. The present study is the first report to examine the association between this polymorphism and hypertension.

### Identification of polymorphism

Genome DNA was extracted using a fully automatic nucleic acid extractor (MagExtractor Genome, Toyobo Co., Ltd., Osaka, Japan). The extracted genome DNA was stored at -20°C until genotyping. The polymerase chain reaction confronting two pair primers method (PCR-CTPP)^22^ was used to analyze the Ala288Ser polymorphism in the ET-1 gene. The design of 4 primers for PCR-CTPP in the franking region of the G/T polymorphism associated with the Ala288Ser polymorphism in the ET-1 gene was as follows: the forward primer was 5′-CCT CGC TCC CAT TCT AAG CAT AGG G-3′, the reverse primer was 5′-CCT TTG CCA GTC AGG AAC CA-3′, the G allele-specific forward primer was 5′-GAT CCC AAG CTG AAA GGC AAG-3′, and the T allele-specific reverse primer was 5′-TCA CAT AAC GCT CTC TGG AGG GA-3′. PCR was performed in a thermal cycler (i-cycler, Bio-rad., CA, USA). The total volume of PCR reaction solution was 10 ml for each sample, containing 2 mmol/L of each primer. The solution contained 10 mmol/L Tris-HCl (pH 8.3), 50 mmol/L KCl, 1.5 mmol/L MgCl2, 250 mmol/L of each dNTP, and 5U of AmpliTaq Gold DNA polymerase (Applied Biosystems, New Jersey, USA). For the PCR procedure, initial denaturation for 10 min at 95°C was subsequent to a hot start at 95°C. Following the PCR cycle with denaturation for 30 sec at 95°C, annealing for 30 sec at 65°C, and polymerization for 60 sec at 72°C was performed 35 times. Samples for electrophoresis were prepared with 4.0 ml of PCR product and 0.4 ml of Loading Buffer (Wako Pure Chemical Industries, Ltd., Osaka, Japan). The samples were electrophoresed in 6% polyacrylamide gels and DNA was visualized by ethidium bromide staining for genotype assessment.

### Statistical analysis

The SAS^®^ software (SAS Institute, Cary, NC) was employed for all statistical analysis. Unpaired analysis of results by a Wilcoxon rank sum test was performed to assess whether differences of quantitative variables between normotensives and hypertensives were significant. Chi-square tests were calculated to evaluate the distribution and trend of sex, lifestyle and genotype. The odds ratios (OR) and 95% confidence intervals (CI) of each factor for incidence of hypertension were estimated by univariate and multivariate unconditional logistic regression analysis. Statistical significance was defined as p<0.05.

### Ethical issue

The present study was approved by the Ethics Committee of the Faculty of Medicine, Tottori University (No.81. 2000, and No.128. 2001).^23^^-^^27^ Informed consent of gene analysis was obtained from each study subject.

## RESULTS

Sex distribution, mean value and standard deviation (SD) of age and various quantitative variables, and distribution of lifestyle factors at the health checkups in 1992 by incidence of hypertension in 1998 are shown in Table 1. The proportion of incident cases of hypertension was larger in males than in females. Mean values of age, BMI, TC, and FBS were higher in hypertensives than in normotensives, respectively. The proportions of incident cases of hypertension were larger in alcohol drinkers and smokers than in nondrinkers and nonsmokers. Similar trends were observed in subjects’ age under 40 years, and 40+, respectively.

**Table 1.** Characteristics of study cohort: measurements in 1992 health checkups.

	All Subjects	Normotensive in 1998	Incident cases of Hypertension	p
Total				

No. of Subjects	922	789	133	
Sex; M/F	540 ( 58.6%)	447 ( 82.8%)	93 ( 17.2%)	0.0055
	382 ( 41.4%)	342 ( 89.5%)	40 ( 10.5%)	< 0.0001
Age; years	37.1 ± 8.5	36.6 ± 8.5	40.2 ± 7.4	0.0003
BMI; kg/m^2^	22.2 ± 2.7	22.1 ± 2.7	23.1 ± 3.0	< 0.0001
TC; mg/dl	190.3 ± 33.5	188.3 ± 32.4	202.2 ± 37.0	0.0045
FBS; mg/dl	93.5 ± 13.5	93.0 ± 11.6	96.7 ± 21.5	
Alcohol Drinking				
Non & Ex.	669 ( 72.6%)	590 ( 88.2%)	79 ( 11.8%)	
Current	253 ( 27.4%)	199 ( 78.7%)	54 ( 21.3%)	0.0004
Smoking				
Non & Ex.	581 ( 63.0%)	513 ( 88.3%)	68 ( 11.7%)	
Current	341 ( 37.0%)	276 ( 80.9%)	65 ( 19.1%)	0.0030

Age 20 to 39 y.o.				

No. of Subjects	501	453	48	
Sex; M/F	318 ( 63.5%)	277 ( 87.1%)	41 ( 12.9%)	
	183 ( 36.5%)	176 ( 96.2%)	7 ( 3.8%)	0.0016
Age; years	30.8 ± 5.9	30.6 ± 5.9	32.2 ± 5.5	0.0874
BMI; kg/m^2^	21.9 ± 2.7	21.7 ± 2.7	23.1 ± 3.0	0.0032
TC; mg/dl	184.2 ± 32.3	182.8 ± 31.8	197.1 ± 33.9	0.0039
FBS; mg/dl	91.4 ± 9.8	91.2 ± 9.9	92.9 ± 8.8	0.0895
Alcohol Drinking				
Non & Ex.	382 ( 76.2%)	351 ( 91.9%)	31 ( 8.1%)	
Current	119 ( 23.8%)	102 ( 85.7%)	17 ( 14.3%)	0.0690
Smoking				
Non & Ex.	292 ( 58.3%)	276 ( 94.5%)	16 ( 5.5%)	
Current	209 ( 41.7%)	177 ( 84.7%)	32 ( 15.3%)	0.0004

Age 40 to 59 y.o.				

No. of Subjects	421	336	85	
Sex; M/F	222 ( 52.7%)	170 ( 76.6%)	52 ( 23.4%)	
	199 ( 47.3%)	166 ( 83.4%)	33 ( 16.6%)	0.1044
Age; years	44.6 ± 3.5	44.5 ± 3.6	44.8 ± 3.4	0.4410
BMI; kg/m^2^	22.6 ± 2.7	22.5 ± 2.6	23.1 ± 3.1	0.1081
TC; mg/dl	197.5 ± 33.5	195.6 ± 31.9	205.0 ± 38.5	0.0692
FBS; mg/dl	96.0 ± 16.6	95.3 ± 13.2	98.8 ± 25.8	0.1897
Alcohol Drinking				
Non & Ex.	287 ( 68.2%)	239 ( 83.3%)	48 ( 16.7%)	
Current	134 ( 31.8%)	97 ( 72.4%)	37 ( 27.6%)	0.0138
Smoking				
Non & Ex.	289 ( 68.6%)	237 ( 82.0%)	52 ( 18.0%)	
Current	132 ( 31.4%)	99 ( 75.0%)	33 ( 25.0%)	0.1258

BMI :body mass index, TC:total cholesterol, FBS:fasting blood sugar.

p : p value for difference between normotensive and hypertensive

p values were determined by chi-square (sex, alcohol drinking, smoking) and Wilcoxon rank sum test (age, BMI, TC, FBS).

The distributions of genotype and allele frequency are shown in Table 2. The relative frequencies of Ala/Ala, Ala/Ser, and Ser/Ser genotypes were 49%, 41%, and 10%, respectively. The allele frequencies were 70% and 30% for Ala and Ser alleles, respectively. These results are consistent with the Hardy-Weinberg equilibrium. Neither the genotype nor the allele frequency showed statistical significance between incidences of hypertension. Similar trends were observed in subjects’ age under 40 years, and 40+, respectively.

**Table 2.** Endothelin Ala288Ser genotypes and allele frequencies by incidence of hypertension.

	normotensive in 1998		Incident cases of Hypertension in 1998		Total		p
Total							

n	789		133		922		
Genotype							
Ala/Ala	385	( 48.8%)	67	( 50.4%)	452	( 49.0%)	
Ala/Ser	324	( 41.1%)	55	( 41.4%)	379	( 41.1%)	
Ser/Ser	80	( 10.1%)	11	( 8.3%)	91	( 9.9%)	0.5774

Ala/Ser + Ser/Ser	404	( 51.2%)	66	( 49.6%)	470	( 51.0%)	0.7361*

Allele frequency							
Ala allele	1094	( 69.3%)	189	( 71.1%)	1283	( 69.6%)	
Ser allele	484	( 30.7%)	77	( 28.9%)	561	( 30.4%)	0.5590

Age 20 to 39 y.o.							

n	453		48		501		
Genotype							
Ala/Ala	220	( 48.6%)	24	( 50.0%)	244	( 48.7%)	
Ala/Ser	190	( 41.9%)	21	( 43.8%)	211	( 42.1%)	
Ser/Ser	43	( 9.5%)	3	( 6.3%)	46	( 9.2%)	0.6359

Ala/Ser + Ser/Ser	233	( 51.4%)	24	( 50.0%)	257	( 51.3%)	0.9703*

Allele frequency					699	( 69.8%)	
Ala allele	630	( 69.5%)	69	( 71.9%)	303	( 30.2%)	0.6352
Ser allele	276	( 30.5%)	27	( 28.1%)			

Age 40 to 59 y.o.							

n	336		85		421		
Genotype							
Ala/Ala	165	( 49.1%)	43	( 50.6%)	208	( 49.4%)	
Ala/Ser	134	( 39.9%)	34	( 40.0%)	168	( 39.9%)	
Ser/Ser	37	( 11.0%)	8	( 9.4%)	45	( 10.7%)	0.7059

Ala/Ser + Ser/Ser	171	( 50.9%)	42	( 49.4%)	213	( 50.6%)	0.8075*

Allele frequency							
Ala allele	464	( 69.0%)	120	( 70.6%)	584	( 69.4%)	
Ser allele	208	( 31.0%)	50	( 29.4%)	258	( 30.6%)	0.6971

p : p value for difference between normotensive and hypertensive.

p values were determined by chi-square test.

* : compared to Ala/Ala

The relationships between genotype, allele frequency and incidence of hypertension estimated by unconditional logistic regression analysis are shown in Table 3. The OR of Ala/Ser and Ser/Ser against Ala/Ala by univariate analysis in whole subjects showed a lower trend, but was not significant. The same trend was observed by multivariate analysis adjusted for sex, age, BMI, TC, FBS, alcohol drinking and smoking habits.

**Table 3.** Odds ratios of endothelin gene polymorphism for incidence of hypertension

	Univariate			Multivariate*		
	
	odds ratio	95% confidence interval	p	odds ratio	95% confidence interval	p
Total (n=922)						
Ala/Ala	1.00			1.00		
Ala/Ser	0.98	0.66 - 1.44	0.8996	0.97	0.65 - 1.44	0.8631
Ser/Ser	0.79	0.40 - 1.56	0.4983	0.75	0.37 - 1.51	0.4131

Ala/Ala	1.00			1.00		
Ala/Ser+ Ser/Ser	0.94	0.65 - 1.36	0.7360	0.92	0.63 - 1.35	0.6687

Age 20 to 39 y.o. (n=501)						
Ala/Ala	1.00			1.00		
Ala/Ser	1.01	0.55 - 1.88	0.9669	1.01	0.53 - 1.94	0.9658
Ser/Ser	0.64	0.18 - 2.22	0.4812	0.58	0.16 - 2.11	0.4050

Ala/Ala	1.00			1.00		
Ala/Ser+ Ser/Ser	0.94	0.52 - 1.71	0.8500	0.93	0.50 - 1.72	0.8053

Age 40 to 59 y.o. (n=421)						
Ala/Ala	1.00			1.00		
Ala/Ser	0.97	0.59 - 1.61	0.9172	0.95	0.57 - 1.59	0.8469
Ser/Ser	0.83	0.36 - 1.91	0.6611	0.82	3.49 - 1.91	0.6372

Ala/Ala	1.00			1.00		
Ala/Ser+ Ser/Ser	0.94	0.59 - 1.52	0.8072	0.92	0.57 - 1.50	0.7387

* : adjusted for sex, age, body mass index, total cholesterol, fasting blood sugar, alcohol, and smoking

## DISCUSSION

The distribution of the Ala288Ser polymorphism in the ET-1 gene among Japanese workers was revealed by the present study; the Ala/Ala, Ala/Ser, and Ser/Ser genotypes were in 49%, 41%, and 10% of subjects, respectively. The Ser allele frequency showed a high proportion at 30%. On the other hand, with both univariate and multivariate analysis adjusted for considerable variables, the polymorphism showed no statistical significance for the incidence of hypertension.

As far as we know, seven studies that assessed the relationship between ET-1 polymorphism and hypertension have been published. The first study reported no effect for a Taq1 restriction fragment length polymorphism on hypertension and blood pressure level.^13^ Stevens et al.^14^ and Brown et al.^16^ studied on single base insertion of adenine at position 138 in the ET-1 gene, and showed the association between the polymorphism and DBP among control subjects with normal blood pressure. The remaining four reports studied the Lys198Asn polymorphism in the ET-1 gene. Among them, a study of pregnant women revealed that the Lys/Asn and Asn/Asn genotypes significantly raised SBP compared to the Lys/Lys genotype during pregnancy, and the effect disappeared after delivery.^6^ Other three studies on the polymorphism were population-based.^15^^,^^17^^,^^18^ These three reports demonstrated that a steeper increase in blood pressure with BMI occurred in obese subjects with this polymorphism, without distinction of race.

There have been no reports available on the Ala288Ser polymorphism in the ET-1 gene, which we studied here, yet. A high proportion of 30% of the Ser allele frequency in this polymorphism was observed. Although no association between the polymorphism and incidence of hypertension was observed after adjusting for confounding factors including lifestyle variables, additional studies are needed to examine the mean of this polymorphism.

The relationship was analyzed, and adjusted for FBS and lifestyle variables as well as sex, age, BMI, and TC, in this study. Participation of insulin in the modulation of plasma ET-1 levels was suspected.^28^^,^^29^ It is also known that a higher concentration of serum insulin is observed in obese subjects.^30^^,^^31^ Thus, FBS was included in the analysis to adjust for the confounding factor of insulin resistance of subjects. Moreover, in the epidemiologic knowledge, chronic exposure to smoking would not increase the risk of incidence of hypertension.^32^ Regarding plasma ET-1 levels, some reports have demonstrated an increase by smoking,^33^^,^^34^ whereas another has reported a decrease.^35^ On the other hand, alcohol drinking has been shown to increase the risk of incidence of hypertension.^36^ Regarding plasma ET-1 levels, one study has demonstrated no induction by alcohol drinking.^37^ The polymorphism studied in this report did not seem to be a candidate gene responsible for incidence of hypertension because it showed no significant relationship after adjusting for these lifestyle variables.

All the reports which have studied the relationship between polymorphism in the ET-1 gene and hypertension were analyzed using case-control association studies. A retrospective cohort study among workers, not hospital-based, was employed in this study. Although a cohort study was employed to reduce the effect of biases, compared to the case-control study, some limitations remain in this investigation. First, for the incident cases of hypertension in this study, we are not able to deny the possibility that they included cases of secondary hypertension. However, the influence of this limitation is thought to be small, since more than 90% of Japanese hypertension cases are essential hypertension.^38^ Second, the influence of our definition of incidence of hypertension. Although 133 subjects developed hypertension in 1998, many of them were assessed based on their blood pressure and showed mild hypertension. The Blood pressure was measured carefully, however, because of the individual variation and other biases^39^ in blood pressure, there is possibility that our definition of incidence of hypertension leads the overestimation of the number of incident cases and results the underestimation of the relationship between the polymorphism and incidence of hypertension. Third, the relationships among study subjects were not confirmed. The possibility that relations and siblings showed similar characteristics and influenced the results cannot be denied. Fourth, the accuracy of the record of health checkups is listed. Lifestyle variables were surveyed, through interview by public health nurses, based on a self-statement by each subject. The presence of recall bias is suspected. A prospective cohort study should be performed for a more exact evaluation.

In our results, in neither univariate analysis nor multivariate analysis after adjusting for considerable variables did the Ala288Ser polymorphism in the ET-1 gene show a statistically significant relationship with incidence of hypertension. The Ala288Ser polymorphism in the ET-1 gene did not seem to be a candidate gene responsible for incidence of hypertension among these study subjects.

## References

[1] Yanagisawa M, Kurihara H, Kimura S, Tomobe Y, Kobayashi M, Mitsui Y, . A novel potent vasoconstrictor peptide produced by vascular endothelial cells. Nature 1988;332:411-5. 2451132 10.1038/332411a0

[2] Levin ER. Endothelins. N Engl J Med 1995;333:356-63. 7609754 10.1056/NEJM199508103330607

[3] Inoue A, Yanagisawa M, Kimura S, Kasuya Y, Miyauchi T, Goto K, . The human endothelin family: three structurally and pharmacologically distinct isopeptides predicted by three separate genes. Proc Natl Acad Sci USA 1989;86:2863-7. 2649896 10.1073/pnas.86.8.2863 PMC287019

[4] Masaki T. Possible role of endothelin in endothelial regulation of vascular tone. Ann Rev Pharmacol Toxicol 1995;35:235-55. 7598493 10.1146/annurev.pa.35.040195.001315

[5] Ross R. The pathogenesis of atherosclerosis: a perspective for the 1990s. Nature 1993;362:801-9. 8479518 10.1038/362801a0

[6] Barden AE, Herbison CE, Beilin LJ, Michael CA, Walters BN, Van Bockxmeer FM. Association between the endothelin-1 gene Lys198Asn polymorphism blood pressure and plasma endothelin-1 levels in normal and pre-eclamptic pregnancy. J Hypertens 2001;19:1775-82. 11593097 10.1097/00004872-200110000-00011

[7] Shichiri M, Hirata Y, Ando K, Emori T, Ohta K, Kimoto S, . Plasma endothelin levels in hypertension and chronic renal failure. Hypertension 1990;15:493-6. 2185151 10.1161/01.hyp.15.5.493

[8] Sarman B, Farkas K, Toth M, Somogyi A, Tulassay Z. Circulating plasma endothelin-1, plasma lipids and complications in Type 1 diabetes mellitus. Diabetes Nutr Metab 2000;13:142-8. 10963390

[9] Hayness WG, Webb DJ. Contribution of endogenous generation of endothelin-1 to basal vascular tone. Lancet 1994;344:852-4. 7916401 10.1016/s0140-6736(94)92827-4

[10] Krum H, Viskoper RJ, Lacourciere Y, Budde M, Charlon V. The effect of an endothelin - receptor antagonist, bosentan, on blood pressure in patients with essential hypertension. Bosentan Hypertension Investigators. N Engl J Med 1998;338:784-90. 9504938 10.1056/NEJM199803193381202

[11] Bloch K, Friedrich S, Lee M, Eddy R, Shows T, Quertermous T. Structural organization and chromosomal assignment of the gene encoding endothelin. J Biol Chem 1989;264:10851-7. 2659594

[12] Inoue A, Yanagisawa M, Takuwa Y, Mitsui Y, Kobayashi M, Masaki T. The human preproendothelin-1 gene. Complete nucleotide sequence and regulation of expression. J Biol Chem 1989;264:14954-9. 2670930

[13] Berge KE, Berg K. No effect of a Taq1 polymorphism in DNA at the endothelin I (EDN1) locus on normal blood pressure level or variability. Clin Genet 1992;41:90-5. 1347490 10.1111/j.1399-0004.1992.tb03640.x

[14] Stevens PA, Brown MJ. Genetic variability of the ET-1 and the ETA receptor genes in essential hypertension. J Cardiovasc Pharmacol 1995;26 Suppl 3:S9-S12. 8587478

[15] Tiret L, Poirier O, Hallet V, Mcdonagh TA, Morrison C, McMurray JJV, . The Lys198Asn polymorphism in the endothelin-1 gene is associated with blood pressure in overweight people. Hypertension 1999;33:1169-74. 10334806 10.1161/01.hyp.33.5.1169

[16] Brown MJ, Sharma P, Stevens PA. Association between diastolic blood pressure and variants of the endothelin-1 and endothelin-2 genes. J Cardiovasc Pharmacol 2000;35 Suppl 2:S41-S43. 10976780 10.1097/00005344-200000002-00010

[17] Asai T, Ohkubo T, Katsuya T, Higaki J, Fu Y, Fukuda M, . Endothelin-1 gene variant associates with blood pressure in obese Japanese subjects. The Ohasama Study. Hypertension 2001;38:1321-4. 11751711 10.1161/hy1101.095333

[18] Jin JJ, Nakura J, Wu Z, Yamamoto M, Abe M, Tabara Y, . Association of endothelin-1 gene variant with hypertension. Hypertension 2003;41:163-7. 12511547 10.1161/01.hyp.0000043680.75107.cf

[19] Kozak M, Holla LI, Krivan L, Vasku A, Sepsi M, Borivoj S, . Endothelin-1 gene polymorphism in the identification of patients at risk for malignant ventricular arrhythmia. Med Sci Monit 2002;8:BR164-BR167. 12011762

[20] Hirakawa M, Tanaka T, Hashimoto Y, Kuroda M, Takagi T, Nakamura Y. JSNP: a database of common gene variations in the Japanese population. Nucl Acid Res 2002;30:158-62. 10.1093/nar/30.1.158 PMC99126 11752280

[21] Tochikubo O. Blood pressure. In: The association for cerebro-cardiovascular disease control. editors. Manual for prevention, control, treatment of cerebro-cardiovascular disease. Tokyo; Hokendojinsya; 2003, p.84-7. (in Japanese)

[22] Hamajima N, Saito T, Matsuo K, Kozaki K, Takahashi T, Tajima K. Polymerase chain reaction with confronting two-pair primers for polymorphism genotyping. Jpn J Cancer Res 2000;91:865-8. 11011111 10.1111/j.1349-7006.2000.tb01026.x PMC5926438

[23] Okada H, Suyama A, Osaki Y, Okamoto M, Yamamoto T, Nanba E, . Association of the Trp64Arg mutation of the *β*3-adrenergic receptor with diabetes mellitus, impaired glucose tolerance and lifestyle in Japanese workers. Yonago Acta Med 2001;44:55-9.

[24] Kishimoto T, Suyama A, Osaki Y, Miyamoto T, Igarashi A, Okamoto M, . A molecular variant of the angiotensinogen gene and hypertension in a case-control study in Japanese. Yonago Acta Med 2001;44:79-83.

[25] Kishimoto T, Suyama A, Igarashi A, Osaki Y, Okamoto M, Yamamoto T, . Angiotensinogen gene variation and hypertension in a cohort study in Japanese. J Epidemiol 2001;11:115-9. 11434422 10.2188/jea.11.115 PMC11701266

[26] Igarashi A, Osaki Y, Okamoto M, Kishimoto T. Association of the endothelial constitutive nitric oxide synthase (eNOS) gene polymorphism and the indices of atherosclerosis. Jpn J Hyg 2001;56:393. (in Japanese)

[27] Kishimoto T, Misawa Y, Kaetsu A, Nagai M, Osaki Y, Okamoto M, . eNOS Glu298Asp polymorphism and hypertension in a cohort study in Japanese. Prev Med. (in press) 10.1016/j.ypmed.2004.03.030 15475025

[28] Wolpert HA, Steen SN, Istfan NW, Simonson DC. Insulin modulates circulating endothelin-1 levels in humans. Metabolism 1993;42:1027-30. 8345807 10.1016/0026-0495(93)90018-j

[29] Ferri C, Bellini C, Desideri G, Di Francesco L, Baldoncini R, Santucci A, . Plasma endothelin-1 levels in obese hypertensive and normotensive men. Diabetes 1995;44:431-6. 7698512 10.2337/diab.44.4.431

[30] Chien KL, Lee YT, Sung FC, Hsu HC, Su TC, Lin RS. Hyperinsulinemia and related atherosclerotic risk factors in the population at cardiovascular risk: a community-based study. Clin Chem 1999;45:838-46. 10351993

[31] Lakka HM, Salonen JT, Tuomilehto J, Kaplan GA, Lakka TA. Obesity and weight gain are associated with increased incidence of hyperinsulinemia in non-diabetic men. Horm Metab Res 2002;34:492-8. 12384825 10.1055/s-2002-34788

[32] Green MS, Jucha E, Luz Y. Blood pressure in smokers and nonsmokers : epidemiologic findings. Am Heart J 1986;111:932-40. 3706114 10.1016/0002-8703(86)90645-9

[33] Goerre S, Staehli C, Shaw S, Luscher TF. Effect of cigarette smoking and nicotine on plasma endothelin-1 levels. J Cardiovasc Pharmacol 1995;26 Suppl 3:S236-S238. 8587374

[34] Wright JL, Tai H, Dai J, Churg A. Cigarette smoke induces rapid changes in gene expression in pulmonary arteries. Lab Invest 2002;82:1391-8. 12379773 10.1097/01.lab.0000032806.45023.08

[35] Barbera JA, Peinad VI, Santos S, Ramirez J, Roca J, Rodriguez-Roisin R. Reduced expression of endothelial nitric oxide synthase in pulmonary arteries of smokers. Am J Respir Crit Care Med 2001;164:709-13. 11520741 10.1164/ajrccm.164.4.2101023

[36] Ohmori S, Kiyohara Y, Kato I, Kubo M, Tanizaki Y, Iwamoto H, . Alcohol intake and future incidence of hypertension in a general Japanese population: the Hisayama study. Alcohol Clin Exp Res. 2002;26:1010-6. 12170111 10.1097/01.ALC.0000021147.31338.C2

[37] Zilkens RR, Rich L, Burke V, Beilin LJ, Watts GF, Puddey IB. Effects of alcohol intake on endothelial function in men: a randomized controlled trial. J Hypertens 2003;21:97-103. 12544441 10.1097/00004872-200301000-00019

[38] Omae T. Essential Hypertension. In: Ueda H. et al. editors. Internal Medicine. 3rd edition. Tokyo; Asakura syoten;1984, p.312-5. (in Japanese)

[39] Asai Y, Ishikawa S, Kayaba K, Goto T, Nago N, Kario K, . Prevalence, awareness, treatment, and control of hypertension in Japanese rural communities. Jpn J Public Health 2001;48:827-36. 11725526

